# Polygenic risk score translation across diverse populations

**DOI:** 10.3389/fcvm.2026.1870807

**Published:** 2026-06-18

**Authors:** Jose E. Krieger

**Affiliations:** Instituto do Coração do Hospital das Clínicas da Faculdade de Medicina da Universidade de São Paulo (InCor-HCFMUSP), São Paulo, Brazil

**Keywords:** admixed populations, ancestry-aware prediction, cardiovascular precision medicine, clinical translation, coronary artery disease, polygenic risk score

## Abstract

Polygenic risk scores (PRSs) are increasingly being considered as tools to refine risk stratification in cardiovascular and cardiometabolic disease, but their clinical translation remains constrained by a central limitation: most currently available PRSs were derived in predominantly European-ancestry datasets and perform less well in admixed and underrepresented populations. This limitation reflects differences in allele frequencies, linkage disequilibrium structure, imputation performance, ancestry-specific effect sizes, and environmental context, and is especially consequential in recently admixed populations, in whom local ancestry and internal heterogeneity further complicate prediction. In this review, we examine recent methodological and translational advances in PRS development across diverse populations, with emphasis on coronary artery disease (CAD), blood pressure and hypertension, type 2 diabetes, obesity, and atrial fibrillation. We highlight the transition from single-ancestry prediction to multi-ancestry frameworks, as well as emerging approaches tailored to admixed genomes, including ancestry deconvolution-based and local-ancestry-aware models. Across traits, broader discovery resources and ancestry-aware methods have improved predictive performance beyond naive European transfer, but progress remains uneven. CAD currently represents the most mature phenotype, with the strongest evidence for clinically relevant gains from multi-ancestry PRS development and validation. Blood pressure and hypertension, as well as type 2 diabetes, show substantial methodological progress but remain limited by calibration, context dependence, and incomplete evidence for implementation. Obesity and atrial fibrillation are advancing rapidly, but their translational readiness remains less developed. We argue that admixed and underrepresented populations should not be viewed only as settings in which PRSs underperform, but as essential contexts for building more robust and clinically generalizable models. The next phase of precision cardiovascular medicine will depend not simply on improving prediction, but on demonstrating that PRS-informed risk assessment can be calibrated, interpretable, and clinically useful across the diverse populations in whom it is intended to guide care.

## Introduction

### Why diversity is central to the future of PRS science

Polygenic risk scores (PRSs) are increasingly being considered for risk stratification in coronary artery disease (CAD), hypertension, type 2 diabetes (T2D), obesity, and atrial fibrillation (AF). However, the field has long been constrained by a structural imbalance: most PRSs were first developed in predominantly European-ancestry datasets and only later tested for transferability to other populations. Because PRS performance depends on allele frequencies, linkage disequilibrium structure, imputation quality, ancestry-specific effect sizes, and environmental context, this design has predictably limited performance in underrepresented populations (4, 5). Landmark work helped define the scale of this problem. Martin et al. showed that then-current PRSs were markedly more accurate in European than non-European populations, with relative prediction accuracy reported as 1.6-fold lower in Hispanic/Latino individuals, 1.7-fold lower in South Asians, 2.5-fold lower in East Asians, and 4.9-fold lower in Africans, on average (1). Later studies reinforced that portability is not simply a between-group problem: Wang et al. quantified the expected decline in predictive accuracy with increasing genetic divergence (2), and Ding et al. showed that polygenic score accuracy can decrease continuously along the ancestry continuum even within conventionally labeled populations (3).

**Figure 1:**
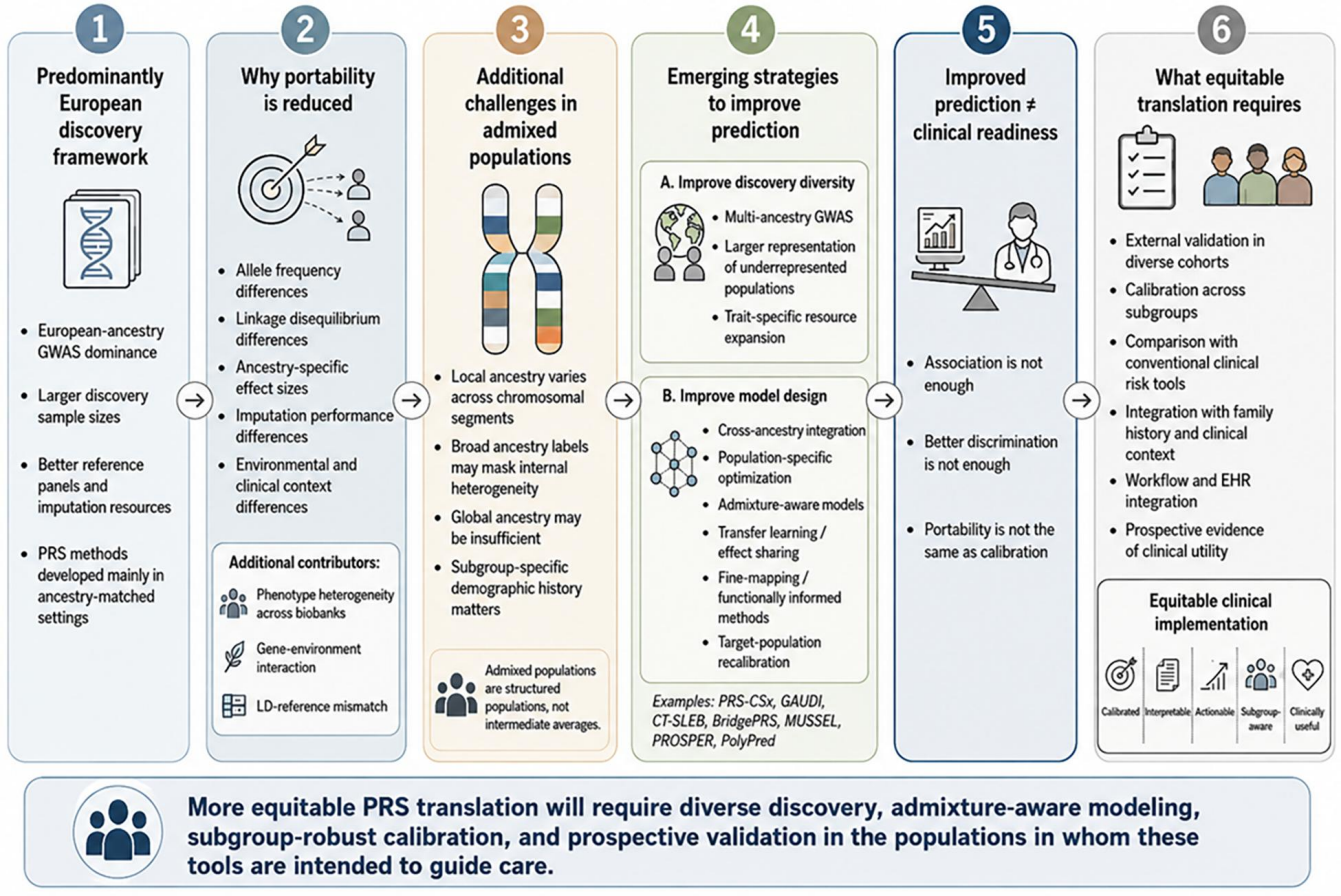
Determinants of PRS portability in admixed and underrepresented populations and the path toward equitable clinical translation. Most polygenic risk scores (PRSs) have been derived from predominantly European-ancestry genome-wide association studies, limiting transferability across diverse populations. Reduced portability reflects differences in allele frequencies, linkage disequilibrium structure, ancestry-specific effect sizes, imputation performance, and environmental and clinical context, and may be further shaped by phenotype heterogeneity, gene-environment interaction, and LD-reference mismatch. These challenges are amplified in admixed populations, in whom local ancestry varies across chromosomal segments and broad population labels may mask substantial internal heterogeneity. Emerging responses include expansion of multi-ancestry discovery resources, cross-ancestry integration, admixture-aware models, transfer-learning and functionally informed approaches, and target-population recalibration. However, improved prediction should not be conflated with implementation readiness; equitable translation additionally requires external validation, subgroup-aware calibration, comparison with conventional risk tools, workflow integration, and prospective evidence of clinical utility.

This issue is particularly important in admixed populations. Unlike relatively homogeneous continental groups, recently admixed populations carry chromosomal segments inherited from distinct ancestral sources, so PRS performance may depend not only on global ancestry but also on local ancestry and ancestry-differential effects. Latin America provides a particularly informative context for this problem, and Brazil is one of the clearest modern examples because of its large-scale, recent, and heterogeneous admixture involving Indigenous American, European, and African contributions. Recent work on Brazilian population history and health has reinforced how deeply admixture shapes genomic architecture and biomedical interpretation, underscoring that admixed populations should not be viewed as diluted versions of continental reference groups, but as genetically structured populations in their own right (6). The determinants of reduced PRS portability in admixed and underrepresented populations, together with the major emerging methodological responses, are summarized in Figure 1.

In this review, we evaluate the maturity of PRS evidence across cardiovascular and cardiometabolic traits using five domains that are central to equitable translation: (1) diversity of discovery data, (2) gain beyond naive European-to-non-European transfer, (3) evidence in admixed and underrepresented populations, (4) calibration and subgroup robustness, and (5) evidence for clinical integration. This framework is used throughout to distinguish advances in statistical prediction from progress toward clinically meaningful and equitable implementation.

### Approach and scope of this review

This article is a focused narrative review rather than a formal systematic review. To improve transparency, the literature considered for inclusion was identified through targeted searches of the recent PRS and cardiovascular genomics literature, with emphasis on studies published from 2019 onward, while also incorporating older landmark papers when they were necessary to define the portability problem or the conceptual foundations of multi-ancestry prediction. Priority was given to studies that met at least one of the following criteria: (1) development or benchmarking of multi-ancestry or admixture-aware PRS methods; (2) application of PRS to major cardiovascular or cardiometabolic traits relevant to this review; (3) explicit evaluation in admixed, non-European, or otherwise underrepresented populations; or (4) relevance to implementation, calibration, subgroup robustness, or health equity. Because the field remains methodologically heterogeneous, this review does not attempt a formal meta-analysis of performance metrics. Instead, it uses a structured comparative framework to evaluate PRS maturity across traits and translational domains.

## Discussion

### Methodological progress from single-ancestry scores to admixture-aware prediction

The methodological evolution of the field has proceeded along two main fronts. The first has been the move from single-ancestry PRSs toward explicitly multi-ancestry models. A landmark contribution in this area was PRS-CSx, introduced by Ruan and colleagues, which integrates GWAS summary statistics from multiple ancestral groups and improves prediction across diverse populations relative to simpler approaches (5). This framework was important not only because it improved performance, but because it formalized a broader principle: investigators do not need to choose between very large but ancestry-mismatched datasets and smaller but more relevant target-population datasets. Instead, information can be borrowed across ancestries in a principled way.

The second major methodological advance has been the development of models tailored specifically to admixed genomes. Marnetto and colleagues showed that ancestry deconvolution and partial polygenic scores can improve susceptibility prediction in recently admixed individuals, demonstrating that genomic mosaicism itself is informative for risk prediction (7). Sun and colleagues extended this logic with GAUDI, which explicitly models ancestry-differential effects in admixed populations and improves prediction when effect sizes differ across the ancestral backgrounds represented within the same genome (8). Together, these studies make a key conceptual point: improving PRS performance in admixed populations is not simply a matter of adding more samples. It also requires models that better reflect how ancestry is structured across the genome.

### Methodological landscape beyond early multi-ancestry models

The field has now expanded well beyond a small number of flagship methods, but the growing number of approaches can obscure a more important question: under what conditions is each class of method most likely to improve prediction, and what tradeoffs does it introduce? In practice, the answer depends on the ancestry composition of the discovery data, the structure of effect-size sharing across populations, the extent of admixture in the target group, the quality of LD resources, and the translational setting in which the score is ultimately intended to be used.

A first major class of approaches is cross-ancestry integration, exemplified by methods such as PRS-CSx, CT-SLEB, BridgePRS, MUSSELL, and PROSPER (5, 9–12). These methods are most likely to perform well when the trait has a substantial shared polygenic architecture across populations and when information borrowed from larger ancestry groups can compensate for smaller target-population datasets. Their principal advantage is that they can improve prediction in underrepresented populations even when target-specific GWAS remain underpowered. This makes them especially attractive for common cardiometabolic and cardiovascular traits in which discovery resources remain heavily imbalanced. However, their gains may be limited when LD patterns differ sharply across populations, when ancestry-specific effects are substantial, or when the target population is insufficiently represented in the discovery substrate. In those settings, effect sharing becomes less reliable and improved discrimination may still coexist with weak calibration.

A second class comprises admixture-aware approaches, such as ancestry deconvolution frameworks and GAUDI (7, 8). These methods are most likely to be advantageous in recently admixed populations, particularly when local ancestry varies substantially across the genome and when effect sizes differ across ancestral backgrounds within the same individual. Their conceptual strength is that they treat admixed genomes as structured mosaics rather than as points between continental reference groups. This makes them especially relevant in Latin American, African American, and other recently admixed populations in which broad ancestry labels often mask important internal genomic structure. Their main limitation is operational rather than conceptual: these methods are more complex, less standardized, and still relatively uncommon in translational pipelines. Thus, despite their biological appeal, they have not yet been incorporated as routinely as cross-ancestry integration methods in large-scale implementation efforts.

A third class includes transfer-learning and related effect-sharing approaches (15). These methods are especially useful when target-population datasets are modest in size but sufficiently informative to allow model adaptation rather than full re-derivation. In that setting, transfer learning can improve performance by leveraging larger external datasets while still tuning prediction toward the target population. This is particularly promising for underrepresented groups in whom discovery resources are growing but remain inadequate for fully independent PRS development. The tradeoff is that these methods depend on how well the transferred representation maps onto the target population; if the external and target settings differ too much genetically, phenotypically, or environmentally, transfer may be less effective or may even reinforce instability in calibration.

A fourth class includes functionally informed and fine-mapping-informed approaches, such as PolyPred (13). These methods are most likely to help when causal variant localization can reduce reliance on ancestry-specific LD tagging and thereby improve transportability across populations. Their appeal lies in the possibility that a more causal and less purely correlational representation of genetic architecture may travel better across ancestry groups than conventional PRS construction. However, this advantage depends on the quality of fine-mapping, the accuracy of functional priors, and the availability of suitable upstream resources. Accordingly, these methods may be highly informative in well-characterized traits and datasets, but their complexity and data demands may limit immediate routine use in broad translational settings.

A fifth perspective comes from biobank-scale benchmarking and ensemble evaluation (14). These studies do not necessarily introduce a new scoring method, but they are increasingly important because they show that variation across biobanks, phenotype definitions, and validation settings may be as influential as variation across methods. This has a critical implication for translation: a method that appears superior in one benchmarking framework may not be consistently superior across traits, datasets, and implementation environments. In other words, method choice cannot be separated from phenotype definition, cohort architecture, and clinical use case.

Taken together, these comparisons suggest that no single methodological class is uniformly best. Cross-ancestry integration methods are currently the most scalable and broadly deployable when polygenic effects are substantially shared and target-specific discovery remains sparse. Admixture-aware methods are likely to be especially valuable when local ancestry and ancestry-differential effects are central to the prediction problem, but they remain less operationally mature. Transfer-learning approaches are particularly promising for underrepresented populations in an intermediate stage of data availability, where adaptation may outperform both naive transfer and full *de novo* derivation. Functionally informed methods may improve portability when improved causal resolution is achievable, but they come with greater modeling and resource complexity. Benchmarking frameworks remind us that performance is always conditional on the phenotype, cohort, and validation environment. The broader lesson is that equitable PRS science is unlikely to converge on a single universal method; instead, it will require method selection that is matched to the genetic architecture of the trait, the ancestry composition of the available data, and the translational context in which the score is intended to operate.

More recently, federated or distributed development strategies have also emerged as a potentially important direction for equitable implementation, especially in settings where individual-level data sharing is constrained. Their translational promise lies less in a specific gain in predictive performance than in the possibility of enabling broader participation of diverse cohorts in PRS development without requiring full centralization of data. In this sense, they may become enabling infrastructure for equitable PRS science rather than simply another competing method.

This comparative view is important because the translational problem is no longer simply whether a PRS can be improved in diverse populations, but which methodological strategy is most appropriate under which data and population conditions. The key distinction is therefore not only between single-ancestry and multi-ancestry prediction, but between settings dominated by shared polygenic signal, local ancestry structure, limited target-population sample size, incomplete causal resolution, or validation heterogeneity. Framed this way, methodological progress becomes easier to interpret in relation to downstream clinical translation.

### Heritability, genetic architecture, and the ceiling of PRS performance

A related issue that helps interpret differences across traits is heritability and broader genetic architecture. All else being equal, traits with higher SNP-heritability and a substantial common-variant component are more likely to offer greater headroom for PRS performance than traits in which environmental exposures, phenotype heterogeneity, or strong developmental and social influences explain a larger share of risk. This does not mean that highly heritable traits are automatically implementation-ready, or that lower-heritability traits lack clinical value. Rather, it suggests that cross-trait differences in PRS maturity partly reflect differences in the underlying predictability of the phenotype itself. This is especially relevant when comparing CAD and blood pressure, which have shown strong polygenic signal and meaningful multi-ancestry gains, with obesity or context-sensitive T2D phenotypes, in which clinical and environmental modifiers may cap the marginal utility of genetic prediction.

These methodological advances help reframe a central problem in the field. Poor PRS portability in underrepresented populations is not an inevitable biological property; it is partly a consequence of how discovery resources and predictive models have historically been built. Admixed populations are therefore not merely difficult validation cohorts. They are scientifically informative settings in which more generalizable predictors can be developed, as illustrated by the key methodological, resource, and implementation-oriented studies summarized in Table 1.

**Table 1:** Methodological, translational, and implementation-oriented studies relevant to PRS performance in diverse and admixed populations.

Study	Focus	Principal contribution	Relevance for diverse/admixed populations	Main limitation
O’Sullivan et al. (4)	Clinical framework	AHA scientific statement defining evaluation domains beyond association alone	Established the importance of calibration, clinical context, interpretability, and equity for cardiovascular PRS translation	Framework paper; not a derivation study
Lennon et al. (16)	Clinical implementation	Selection and validation of PRSs for implementation in diverse US populations	Linked PRS optimization to pragmatic deployment in heterogeneous populations	Not specifically focused on admixed-genome modeling
Ruan et al. (5)	Multi-ancestry modeling	Developed PRS-CSx for integrating summary statistics across ancestries	Landmark demonstration that cross-ancestry integration improves portability	Does not explicitly model local ancestry
Marnetto et al. (7)	Admixed-genome prediction	Used ancestry deconvolution and partial PRSs in recently admixed individuals	Showed that genomic mosaicism can improve prediction if modeled explicitly	More methodological than translational
Sun et al. (8)	Admixed-genome prediction	Developed GAUDI to model ancestry-differential effects	Major advance for recently admixed populations and local-ancestry-aware prediction	Methodologically complex; limited translational experience
Zhang et al. (9) (CT-SLEB)	Multi-ancestry modeling	Developed a scalable approach combining clumping-thresholding, empirical Bayes, and super learning	Improved non-European prediction while maintaining computational practicality	Still depends on available ancestry-specific discovery resources
Hoggart et al. (10) (BridgePRS)	Cross-ancestry Bayesian modeling	Leveraged shared genetic effects across ancestries to improve PRS portability	Useful when non-European discovery datasets remain smaller than European datasets	Performance depends on the degree of effect sharing across populations
Jin et al. (11) (MUSSELL)	Multi-ancestry Bayesian/ensemble modeling	Borrowed information across ancestry groups through hierarchical modeling and ensemble learning	Highlights flexible ancestry-specific prediction strategies beyond simple transfer	Methodological complexity may limit immediate routine uptake
Zhang et al. (12) (PROSPER)	Ensemble penalized regression	Introduced a multi-ancestry ensemble regression framework with strong performance in simulations and real data	Expands the methodological landscape beyond summary-statistic-only integration	Requires substantial training-data structure and benchmarking
Weissbrod et al. (13) (PolyPred)	Functionally informed/fine-mapping-informed prediction	Leveraged fine-mapping and multipopulation training to improve cross-population prediction	Suggests that more causal modeling may improve portability	Requires complex upstream resources and assumptions
Monti et al. (14)	Biobank-scale benchmarking	Compared multiple PRS methods across five biobanks and showed that variation across biobanks can exceed variation across methods	Emphasizes that phenotype and cohort heterogeneity materially shape PRS performance	Benchmarking study rather than a translational framework
Wu et al. (15)	Transfer learning	Applied transfer-learning principles to improve polygenic score prediction for underrepresented groups	Highlights a major emerging direction for equitable prediction	Early translational implications remain to be defined
Nunes et al. (6)	Population genomics	Characterized Brazilian admixture and its impact on health-related genomic variation	Reinforced the importance of modeling admixed populations as structured populations	Not a PRS method paper
Tcheandjieu et al. (19)	Diverse GWAS resource	Large multi-ancestry CAD GWAS	Expanded the discovery substrate for downstream PRS development	Discovery resource rather than implementation study
Patel et al. (17)	CAD application	Developed GPS_Mult for CAD	Proof-of-principle that multi-ancestry design improves clinically relevant prediction	Performance gaps persisted across ancestries
Smith et al. (18)	CAD application	Multi-ancestry CHD PRS with population-specific optimization	Highlighted the value of ancestry-aware optimization and external validation	Uneven performance remained across groups
Hutten et al. (20)	Latino subgroup evaluation	Compared CHD PRS performance across Hispanic/Latino subgroups	Showed that broad ethnic labels can mask important internal heterogeneity	Recent subgroup-specific evidence; broader replication across Latino subgroups and external cohorts remains needed
Teixeira et al. (23)	Brazilian transferability	Tested European-derived BP PRSs in admixed Brazilian cohorts	Practical example of signal transfer with need for local recalibration	Did not establish full implementation readiness
Roselli et al. (30)	AF resource	Large GWAS/PRS meta-analysis in AF (>180,000 cases)	Major resource for future ancestry-aware AF prediction	AF translation remains earlier than CAD

Table 1. Methodological, translational, and implementation-oriented studies relevant to PRS performance in diverse and admixed populations. The table summarizes key conceptual frameworks, statistical methods, population-genomic resources, benchmark studies, and trait-specific translational applications that shape the current landscape of equitable PRS development and implementation across diverse populations. Together, these studies illustrate that different methodological classes are likely to be advantageous under different combinations of ancestry composition, effect-size sharing, admixture structure, causal resolution, and translational setting.

### Clinical implementation frameworks: moving beyond association alone

As the field has matured, an equally important shift has occurred from pure statistical performance toward clinical implementation frameworks. The American Heart Association scientific statement on cardiovascular PRSs helped define the broader translational agenda by emphasizing that implementation requires more than association or modest gains in discrimination; it also requires calibration, clinical context, interpretability, workflow integration, and equity across populations (4). More recently, Lennon and colleagues operationalized this translational perspective by selecting, optimizing, and validating ten chronic disease PRSs for clinical implementation in diverse US populations, showing that pragmatic deployment requires disease-specific optimization, ancestry-aware evaluation, and explicit attention to performance in real-world heterogeneous cohorts (16).

**Figure 2:**
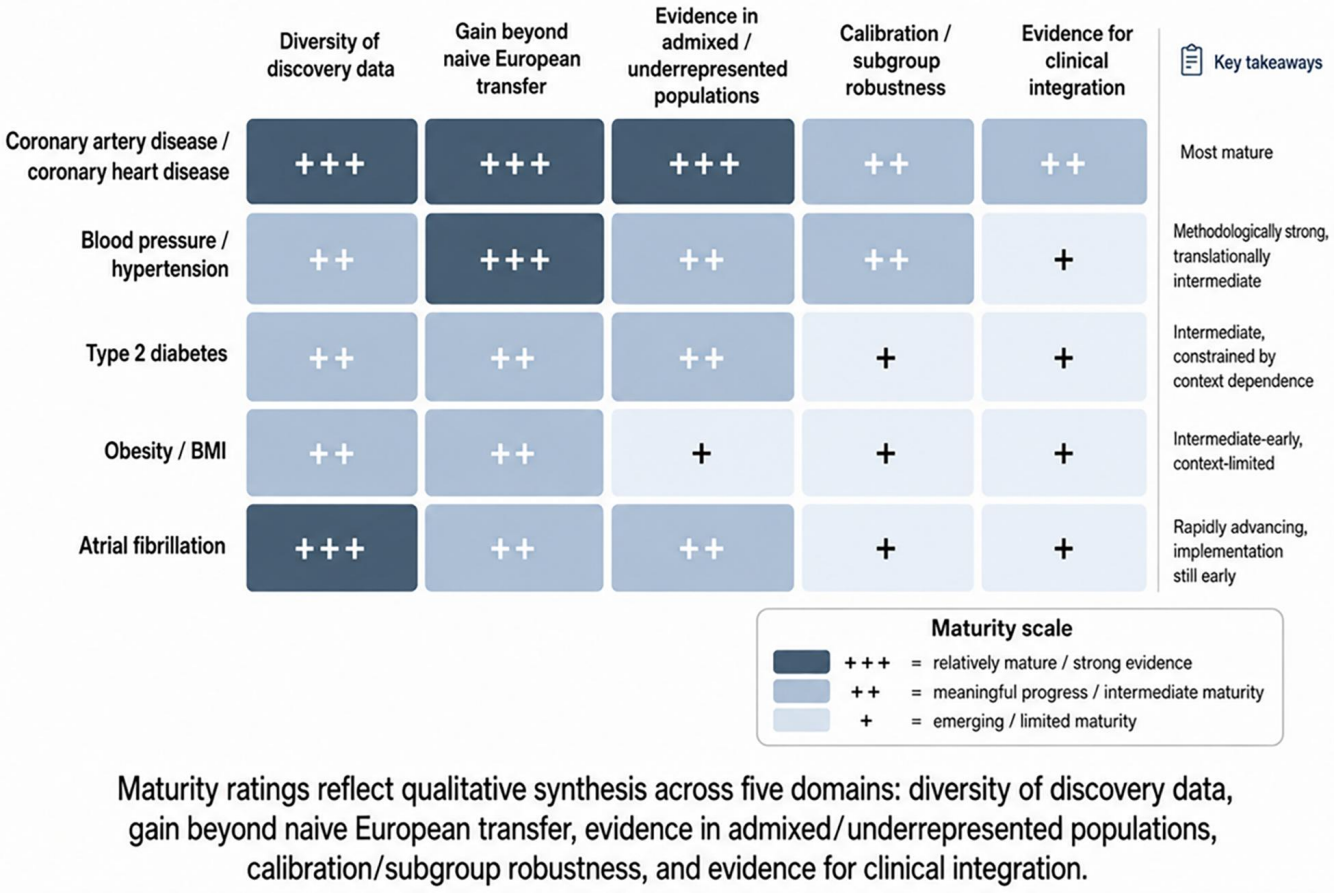
Comparative maturity of PRS evidence across cardiovascular and cardiometabolic traits in diverse populations. The figure compares coronary artery disease/coronary heart disease, blood pressure/hypertension, type 2 diabetes, obesity/body mass index, and atrial fibrillation across five domains central to equitable translation: diversity of discovery data, gain beyond naive European-to-non-European transfer, evidence in admixed and underrepresented populations, calibration/subgroup robustness, and evidence for clinical integration. CAD is shown as the most mature phenotype overall, reflecting the strongest evidence for clinically relevant gains from multi-ancestry PRS development and validation. Blood pressure/hypertension and type 2 diabetes show substantial methodological progress but remain limited by calibration, context dependence, and incomplete implementation evidence. Obesity/body mass index and atrial fibrillation are advancing, but their translational readiness remains less developed. Maturity ratings reflect qualitative synthesis across the five specified domains rather than the number of publications alone.

These implementation-oriented studies are important because they clarify that the goal is not simply to produce ever-larger PRSs, but to identify scores that can be used responsibly in clinical care. In other words, the question is no longer only whether a PRS predicts disease, but whether it improves decision-making in a way that is robust across populations. The relative maturity of evidence across the major cardiovascular and cardiometabolic traits discussed in this review is summarized in Figure 2.

### Quantitative perspective on portability loss and improvement

Because performance metrics vary widely across studies, phenotypes, and validation cohorts, direct cross-trait comparison remains imperfect. Nevertheless, the available literature makes clear that portability losses are often large rather than trivial. Martin et al. showed multi-fold reductions in relative prediction accuracy outside European populations (1), and Wang et al. provided a theoretical and empirical framework showing why this decline is expected with increasing genetic distance between discovery and target populations (2). Ding et al. further demonstrated that accuracy decreases across the ancestry continuum even within nominal ancestry labels, reinforcing the inadequacy of coarse categorization (3). These studies do not imply that all cardiovascular and cardiometabolic traits exhibit identical losses, but they do establish an important baseline: the expected decrement in performance from naive transfer is large enough that modest methodological gains should not automatically be interpreted as clinically meaningful.

### Coronary artery disease: the most mature example of multi-ancestry improvement

The trait-specific sections below apply the framework outlined above comparatively, emphasizing where progress has occurred across discovery, prediction, subgroup performance, and translation, and where important gaps remain.

Among cardiovascular traits, CAD remains the clearest and most mature example of PRS advancement across the five evaluative domains considered in this review, including diversity of discovery data, gain beyond naive European transfer, evidence in admixed populations, subgroup-aware performance assessment, and early but still incomplete movement toward clinical integration. Patel and colleagues developed GPS_Mult, a multi-ancestry CAD PRS that integrated GWAS data across five ancestries for CAD and multiple genetically correlated CAD risk factors. In external validation, GPS_Mult improved prediction across diverse groups, including Hispanic individuals, and outperformed earlier CAD scores more heavily anchored in European discovery data (17). This was a critical study because it demonstrated that diverse discovery and multi-ancestry integration can materially improve prediction for a major cardiovascular disease rather than merely preserve a small transferred signal.

A complementary advance came from Smith and colleagues, who developed a multi-ancestry PRS for coronary heart disease using ancestrally diverse GWAS data and population-specific optimization. Their work showed that multi-ancestry construction generally outperformed ancestry-specific clumping-and-thresholding approaches, while also underscoring that performance remained heterogeneous across ancestry groups (18). This is an important corrective to simplistic narratives of success: the field has clearly moved beyond naive European transfer, but equitable performance across populations has not yet been achieved.

The discovery substrate supporting CAD prediction has also become more diverse. Tcheandjieu and colleagues performed a large-scale GWAS of CAD in genetically diverse populations, expanding the multi-ancestry evidence base for downstream PRS development and improving the foundation for transferability beyond European cohorts (19). This type of work is critical because the predictive ceiling of any PRS depends in part on whether the discovery architecture itself reflects the populations in which the score will eventually be used. In this sense, CAD is the phenotype in which the field has progressed furthest from diverse discovery to improved cross-population prediction, although calibration robustness and implementation readiness still lag behind discovery and score construction.

Studies in Hispanic/Latino populations add an especially important nuance. In the Hispanic Community Health Study/Study of Latinos, PRS performance for coronary heart disease differed across Hispanic/Latino subgroups, consistent with differences in admixture structure between Caribbean and Mainland populations (20). This observation shows that even categories such as “Latino” may conceal substantial internal genomic heterogeneity. The implication is broad, since the relevant unit of PRS evaluation is often more finely structured than a continental or pan-ethnic label.

Taken together, the CAD literature now establishes four points with reasonable confidence. First, diverse GWAS has become a critical discovery enabler, expanding the variant architecture available for downstream prediction and improving the substrate for transferability beyond European cohorts (19). Second, multi-ancestry PRS construction is a real predictive advance, with evidence that integrated models outperform earlier European-centric scores and can improve risk stratification in non-European populations (17, 18). Third, subgroup analyses have shown that heterogeneity within broad categories such as Hispanic/Latino populations is clinically and methodologically relevant, indicating that internal population structure matters for interpretation (20). What remains uncertain is the extent to which these gains translate into consistently well-calibrated, clinically actionable prediction across the full range of admixed and underrepresented populations. Thus, while CAD is clearly the most mature phenotype in this field, true implementation remains incomplete. The next translational stage will require prospective studies demonstrating stable calibration, incremental value over established prevention tools, and measurable influence on real clinical decision-making across diverse care settings.

### Blood pressure and hypertension: a strong model for cross-population optimization

For blood pressure and hypertension, the field is particularly strong in the first two evaluative domains, diversity-aware methodological development and gain beyond naive European transfer, while evidence for calibration robustness and clinical integration remains less mature. Kurniansyah and colleagues developed a multi-ethnic hypertension PRS associated with hypertension prevalence and progression across adulthood, and later showed that PRS-CSx-based blood pressure scores performed best across multiple ethnicity groups (21, 22). Hypertension is a particularly compelling phenotype for translational PRS work because it is common, measurable, clinically actionable, and tightly linked to downstream cardiovascular outcomes.

Evidence from admixed populations reinforces both the promise and the limitations of transferability. In Brazilian cohorts, European-derived systolic blood pressure PRSs retained measurable association with blood pressure and hypertension, showing that predictive signal can transfer into admixed settings (23). At the same time, these results also illustrate a recurring lesson in PRS science: association is not the same as calibration, and signal transfer is not equivalent to clinical readiness. These studies also provide meaningful evidence in admixed and underrepresented populations, but they stop short of demonstrating subgroup-stable calibration or defining implementation-ready thresholds for clinical use.

For blood pressure and hypertension, it is now convincingly established that ancestry-aware and multi-ancestry methods improve prediction beyond naive cross-population transfer, making this one of the methodologically strongest areas in the field (21, 22). The evidence also supports the view that PRS portability for these traits can be meaningfully improved in diverse and admixed populations, including through PRS-CSx-based approaches and local validation in cohorts such as those from Brazil (23). What remains less certain, however, is how these gains should be translated into practice, because the field is still weaker on clinically actionable thresholds, calibration standards, and evidence for management-changing use. In other words, blood pressure genetics is ahead of blood pressure implementation. The next translational step will require studies that move beyond association and discrimination to define whether PRS-informed classification improves earlier identification, treatment targeting, or long-term prevention in a way that is consistent across ancestry groups.

### Type 2 diabetes: progress with a defining context dependence challenge

For type 2 diabetes, the literature supports substantial progress in discovery diversity, trans-ancestry prediction, and cross-population validation, but also shows that PRS maturity is constrained by pronounced context dependence in calibration and interpretation. Mahajan and colleagues showed that diverse populations enhance both locus discovery and translational potential in T2D genetics, helping establish a broader foundation for risk prediction across ancestries (24). Ge and colleagues then developed and validated a trans-ancestry T2D PRS across diverse populations, supporting the idea that clinically relevant genetic stratification for T2D can be improved through ancestry-aware derivation (25). More recently, Guo and colleagues showed that T2D PRS performance is context dependent, varying according to age, sex, obesity, and hypertension (26). This makes T2D especially informative within the present framework, because it illustrates that gain beyond European transfer does not necessarily translate into subgroup-robust performance or immediate clinical integration.

For T2D, the field has established that diverse discovery improves both locus identification and the portability of polygenic prediction, and that trans-ancestry PRSs can outperform more ancestry-restricted approaches (24, 25). It is also increasingly clear that T2D is not simply another example of the CAD story, since here context dependence is not a side issue but a defining challenge, because PRS performance varies with age, sex, obesity, hypertension, and likely other environmental and clinical modifiers (26). What therefore remains uncertain is not only how well a score transfers across populations, but under what clinical and metabolic conditions it remains informative enough to support decision-making. The next translational stage will require integrated evaluations in which ancestry-aware PRSs are assessed jointly with major non-genetic modifiers, with particular emphasis on calibration, subgroup robustness, and incremental value within realistic screening and prevention frameworks.

### Obesity and BMI: progress with persistent environmental and contextual constraints

For obesity and BMI, broader discovery resources have improved polygenic prediction across ancestries, but progress across the five evaluative domains is more uneven than for CAD, particularly in calibration robustness and translational readiness. Smit and colleagues reported large-scale polygenic prediction of BMI and obesity across ancestries and through the life course, showing that broader discovery and larger datasets can materially improve prediction outside European populations (27). Thus, although obesity PRSs clearly benefit from more diverse discovery data and improved cross-population modeling, evidence in admixed populations remains less mature and clinically interpretable performance remains strongly shaped by non-genetic context. At the same time, the uneven performance of BMI PRSs across populations highlights a broader point: portability is not determined only by genomic distance. Traits with strong environmental, developmental, and social-patterning components may remain difficult to predict equitably even when statistical methods improve.

For obesity and BMI, the current evidence convincingly shows that larger and more diverse discovery resources can improve predictive performance across ancestries, and that multi-ancestry scaling is preferable to continued reliance on European-centric derivation (27). At the same time, this is also the trait area in which the limits of PRS portability are especially visible, because strong environmental, developmental, and social-patterning effects constrain how far statistical improvement alone can take the field. What remains uncertain is whether BMI PRSs can achieve the combination of calibration, robustness, and clinical interpretability needed for equitable translational use, particularly in admixed populations with heterogeneous exposures and life-course risk structures. The next translational step will require studies that explicitly test PRS performance within environmental and social context, rather than treating these factors as background noise around a genomic signal.

### Atrial fibrillation: from expanding resources to early translational promise

For AF and arrhythmia, the evidence base has historically been less mature than for CAD, and current progress is strongest in the domains of discovery scale and resource generation rather than in calibration robustness or clinical implementation. Earlier work showed that AF PRSs can add predictive value beyond conventional risk factors in cardiovascular populations, supporting the broader concept that inherited susceptibility contributes meaningfully to AF risk stratification (28). Work in Hispanic/Latino populations also suggested that AF genetic susceptibility can be modulated and stratified in admixed groups, although this literature was smaller and earlier in development (29).

A major recent advance is the large meta-analysis by Roselli and colleagues, published in Nature Genetics in 2025, which examined genome-wide associations and polygenic risk prediction for AF in more than 180,000 cases (30). This study is important not only because of its scale, but because it provides a substantially richer resource for future AF PRS development, benchmarking, and ancestry-aware refinement. In this respect, AF may now be entering a phase analogous to that reached earlier in CAD, where sufficiently large and diverse resources begin to support more credible cross-population optimization. Even so, stronger discovery resources do not by themselves establish mature evidence for subgroup-robust calibration or implementation-ready clinical use.

For AF, the literature now supports the view that inherited susceptibility contributes meaningfully to risk stratification and that the resource base for ancestry-aware prediction has expanded substantially (28). Roselli et al. changes the resource landscape, but not yet the implementation landscape, and the field now has a much stronger foundation for future AF PRS development and benchmarking, but still lacks the same depth of translational evidence that now exists for CAD (30). What remains uncertain is how far these expanded resources will improve calibration, subgroup performance, and actionable prediction in admixed and underrepresented populations (29, 30). The next translational stage will require external validation across diverse cohorts, comparison with conventional AF risk markers, and clearer demonstration that PRS-informed stratification can influence prevention or surveillance in practice.

Trait-specific evidence for PRS development and translational readiness across diverse populations is summarized in Table 2.

**Table 2:** Trait-specific evidence for PRS performance across cardiovascular and cardiometabolic diseases in diverse populations.

Trait	Key studies	Main advance	Evidence in diverse/admixed populations	Main translational barrier	Maturity^a^
Coronary artery disease/coronary heart disease	Patel et al. (17); Smith et al. (18); Tcheandjieu et al. (19); Hutten et al. (20)	Strongest current example of clinically relevant gain from diverse discovery and multi-ancestry PRS development	Robust evidence in multi-ancestry cohorts, including Hispanic/Latino populations; subgroup heterogeneity also demonstrated	Residual ancestry-related performance gaps; incomplete calibration and prospective utility evidence	+++
Blood pressure/hypertension	Kurniansyah et al. (21); Kurniansyah et al. (22); Teixeira et al. (23)	Multi-ancestry and PRS-CSx-based approaches improve prediction across groups	Evidence in multi-ethnic cohorts and admixed Brazilian cohorts	Limited clinically actionable thresholds, incomplete calibration evidence, and limited implementation data	++
Type 2 diabetes	Mahajan et al. (24); Ge et al. (25); Guo et al. (26)	Diverse discovery and trans-ancestry PRSs improve prediction	Good evidence across diverse populations, though less specifically focused on recently admixed groups	Context dependence and difficulty translating cross-population gain into subgroup-robust use	++
Obesity/BMI	Smit et al. (27)	Larger and more diverse datasets improve prediction across ancestries	Broad multi-ancestry evidence, but evidence in admixed populations remains less mature	Strong environmental and developmental constraint on calibration, robustness, and clinical interpretability	++
Atrial fibrillation	Marston et al. (28); Chalazan et al. (29); Roselli et al. (30)	Rapidly expanding resource base for AF genetics and PRS development	Emerging evidence in diverse cohorts, including Hispanic/Latino populations, with a major new cross-population resource	Resource growth exceeds current calibration, validation, and implementation evidence	++

a Maturity scale: +++, relatively mature evidence base with consistent multi-ancestry improvement and plausible translational relevance; ++, meaningful progress with important remaining barriers; +, emerging evidence base with limited translational support. Maturity ratings reflect qualitative synthesis across five domains: diversity of discovery data, gain beyond naive European transfer, evidence in admixed/underrepresented populations, calibration/subgroup robustness, and evidence for clinical integration.

Table 2. Trait-specific evidence for PRS performance across cardiovascular and cardiometabolic diseases in diverse populations. The table summarizes current evidence across the major phenotypes discussed in this review, including key studies, principal advances, evidence in diverse and admixed populations, major translational barriers, and overall maturity. CAD currently represents the most mature case for multi-ancestry PRS translation, whereas blood pressure/hypertension and type 2 diabetes show substantial progress with persistent limitations in calibration and implementation. Obesity/body mass index and atrial fibrillation remain less translationally mature despite important recent advances.

### Clinical translation: from statistical performance to equitable use

The translational challenge for PRS is no longer mainly statistical. It is increasingly clinical, operational, and ethical. A PRS may improve discrimination and yet still perform poorly in practice if it is not well calibrated in specific ancestry groups, social contexts, or health-system settings. This distinction is important because clinical deployment depends not only on rank ordering, but on estimating risk accurately enough to guide interventions. In underrepresented and admixed populations, calibration errors may have especially important consequences because they can shift eligibility for prevention, screening, or treatment in already underserved groups (4, 16).

A related issue is how PRSs are positioned in care. For common cardiometabolic and cardiovascular diseases, the most plausible role for PRS is not as a stand-alone diagnostic tool, but as a risk enhancer layered onto conventional prevention frameworks. In CAD, hypertension, and possibly T2D, PRSs may help identify individuals whose inherited susceptibility is not fully captured by short-term clinical scores, family history, or standard biomarkers. But that role requires more than statistical association. It requires subgroup-specific validation, recalibration, workflow integration, and ideally prospective evidence that PRS-informed care improves management (4).

### Current clinical implementation and near-term use cases

Although broad clinical deployment remains premature, PRS implementation is no longer purely hypothetical. Recent work has moved toward health-system-oriented PRS reporting and implementation frameworks in which genomic risk is integrated with conventional clinical assessment rather than treated as a stand-alone result (16, 31). These advances suggest that the field is entering an early implementation phase, but also reinforce that operational feasibility is not the same as equitable readiness across populations.

The most realistic near-term role for PRS in precision cardiovascular medicine is likely to be selective rather than population-wide. In CAD, PRS may be most useful in intermediate-risk adults, where inherited risk could help refine decisions when conventional prevention thresholds remain uncertain. It may also be particularly informative in younger adults, whose short-term absolute risk often appears low despite substantial genetic susceptibility, especially when interpreted together with family history. More broadly, PRS could support prioritization of longitudinal prevention intensity, helping identify individuals for whom earlier or more sustained intervention may be justified over the life course. These applications are most plausible in care settings already equipped for genomic testing, structured clinical risk assessment, and electronic health record integration, where PRS can function as an interpretable risk-enhancing component of a broader prevention framework. In diverse and admixed populations, however, even these targeted use cases will depend on demonstration that PRS-informed decisions remain appropriately calibrated across ancestry groups and clinical contexts.

These potential use cases, however, also make clear the need to define where current PRS frameworks remain insufficient and where translational claims should still be considered provisional.

### Failure modes and boundaries of current PRS translation

Despite the progress outlined above, several failure modes continue to limit the responsible use of PRSs in cardiovascular and cardiometabolic medicine. One of the most important is the tendency to equate statistical association with clinical readiness. A score may show reproducible association with disease, or even modest gains in discrimination, yet still fail to provide sufficiently stable calibration, subgroup robustness, or incremental value over established clinical tools to justify use in practice. This distinction is especially important in admixed and underrepresented populations, in whom errors in calibration may have disproportionate clinical consequences (4, 16).

A second boundary is the continued reliance on broad ancestry or ethnic labels as proxies for genomic structure. Categories such as “Latino,” “Hispanic,” or even “African ancestry” are often too coarse to capture the internal heterogeneity that shapes PRS performance. In admixed populations, local ancestry, subgroup-specific demographic history, and cohort-specific environmental context may all influence prediction in ways that are obscured by broad labels. As a result, apparent validation in a heterogeneous category may overstate portability and mask clinically relevant subgroup differences (20).

A third limitation is that local-ancestry-aware and admixture-aware methods are not yet routine in translational pipelines. Although approaches such as ancestry deconvolution and GAUDI represent important advances, they remain methodologically more complex than the approaches most commonly used in large-scale PRS construction and evaluation. This means that the populations in whom these methods are most needed are often still being assessed with models that only partially capture their underlying genomic structure (7, 8).

In addition to ancestry mismatch and local ancestry effects, other contributors to reduced portability deserve explicit attention. Phenotype heterogeneity across biobanks can alter both effect estimation and apparent transferability. Gene-environment interaction can change the meaning of a given polygenic signal across cohorts with different exposure structures. PRS performance may also vary according to the LD reference panels used for effect estimation and downstream scoring, especially when discovery and target populations differ substantially (14). These issues reinforce that reduced portability is a multidimensional problem that cannot be solved by ancestry-aware modeling alone.

A fourth failure mode is the assumption that demonstration of signal transfer in an admixed or underrepresented cohort is sufficient evidence for implementation. In reality, evidence that a PRS is associated with a trait in such a cohort should be viewed as an early step rather than an endpoint. Translation requires additional evidence of calibration, subgroup stability, clinical interpretability, and utility in decision-making. Without these elements, there is a risk that PRSs will be introduced into practice in ways that appear genomically sophisticated but are not yet sufficiently reliable for equitable care.

Taken together, these boundaries do not diminish the value of PRS research in diverse populations; rather, they define the conditions under which progress should be interpreted responsibly. The next phase of the field will depend not only on improving prediction, but also on recognizing where current methods remain insufficient and where claims of readiness should remain provisional.

### Social determinants of health and equitable translation

Equitable translation cannot be understood through genomic architecture alone. Social determinants of health, access to care, and cohort-specific exposure structure shape the interpretation and downstream consequences of genetic risk prediction across populations. For PRS research in diverse populations, this means that calibration, portability, and fairness should increasingly be considered alongside non-genetic determinants of health rather than in isolation. Incorporating these dimensions is likely to be essential if multi-ancestry PRS frameworks are to become not only more accurate, but also more equitable in practice (32).

### Why admixed and underrepresented populations are central, not peripheral

Admixed and underrepresented populations are particularly important in this translational landscape because they expose the limits of oversimplified assumptions about ancestry and portability. At the same time, they provide a scientific opportunity. By stress-testing PRS models under realistic genomic heterogeneity, they can reveal failure modes that remain hidden in more homogeneous cohorts. Their haplotypic diversity and ancestry mosaics can also improve fine-mapping, illuminate local ancestry effects, and clarify population-specific transferability (6–8). Brazil provides a particularly informative illustration of this principle, but the broader lesson extends far beyond any single population, as admixed and underrepresented groups are essential to the development of genuinely generalizable models rather than afterthoughts in PRS science.

## Conclusions

The PRS field has moved decisively beyond the stage at which reduced performance outside European populations could be regarded as a secondary concern. Multi-ancestry GWAS, ancestry-aware modeling, admixture-informed methods, and implementation-oriented validation studies now show that more equitable prediction is achievable, although important gaps remain. At the same time, progress is uneven across traits and across the domains most relevant to translation. CAD remains the most mature example of clinically relevant progress, with the strongest evidence for gains from diverse discovery, multi-ancestry score construction, and validation beyond naive European transfer. Blood pressure and T2D provide strong complementary models, but both remain constrained by incomplete calibration and limited implementation evidence. Obesity underscores the persistent importance of environmental and clinical context, whereas AF is advancing rapidly with the expansion of large-scale discovery and benchmarking resources but has not yet reached the same translational maturity as CAD. Across traits, the central lesson is clear: underrepresented and admixed populations are not peripheral to the future of PRS science, they are essential to it. Future progress will depend not only on expanding diverse discovery resources, but also on matching methodological strategy to the underlying trait architecture, ancestry structure, and intended clinical use case. The next phase of the field will depend not only on improving prediction, but on demonstrating that PRS-informed risk assessment can be calibrated, subgroup-robust, and clinically useful in the diverse populations in whom it is intended to guide care.

## Clinical perspective

### What is new?

- This review synthesizes recent progress in polygenic risk score (PRS) development across diverse populations with a specific focus on cardiovascular and cardiometabolic traits.
- It applies a comparative framework to assess PRS maturity across five domains relevant to clinical translation: diversity of discovery data, gain beyond naive European transfer, evidence in admixed and underrepresented populations, calibration/subgroup robustness, and evidence for clinical integration.
- It identifies coronary artery disease as the most mature use case for multi-ancestry PRS translation, while showing that blood pressure/hypertension, type 2 diabetes, obesity, and atrial fibrillation remain at different and generally less advanced translational stages.
- It highlights that improved prediction in diverse populations requires not only broader discovery resources, but also ancestry-aware and admixture-informed methods.
- It emphasizes that admixed and underrepresented populations are not peripheral to PRS science, but essential to the development of more generalizable and clinically credible prediction frameworks.

### What are the clinical implications?

- Polygenic risk scores are unlikely to be most useful as stand-alone tests; their most plausible near-term role is as risk-enhancing tools within broader cardiovascular prevention frameworks.
- In coronary artery disease, PRSs may be most informative in intermediate-risk adults and in younger individuals whose short-term risk appears low despite substantial inherited susceptibility, particularly when interpreted alongside family history and other clinical markers.
- Translation into practice will require more than improved discrimination; subgroup-specific calibration, robustness across admixed and underrepresented populations, and prospective evidence of clinical utility are essential before broader implementation can be justified.
- For health systems already equipped for genomic testing and electronic health record integration, PRSs may help support more individualized prevention intensity, but only if their performance remains interpretable and equitable across diverse populations.
- More broadly, this review suggests that equitable cardiovascular precision medicine will depend on treating diversity as a foundational design principle for discovery, validation, and clinical deployment rather than as a *post hoc* consideration.
